# Histological transformation into SCLC: An important resistance mechanism of NSCLC upon immunotherapy

**DOI:** 10.3389/fimmu.2023.1275957

**Published:** 2023-10-30

**Authors:** Jiao Zeng, Xinjing Ding, Jianghua Ding, Xin Wang

**Affiliations:** ^1^ Department of Hematology & Oncology, Jiujiang University Affiliated Hospital, Jiujiang, Jiangxi, China; ^2^ Graduate Department, Gannan Medical University, Ganzhou, Jiangxi, China; ^3^ Department of Oncology, First Affiliated Hospital of Nanchang University, Nanchang, Jiangxi, China; ^4^ Department of Social Medicine and Public Health, School of Basic Medicine, Jiujiang University, Jiujiang, Jiangxi, China

**Keywords:** histological transformation, small cell lung cancer, resistance, immunotherapy, non-small cell lung cancer

## Abstract

The phenomenon of histological transformation has been widely reported in advanced non-small cell lung cancer (NSCLC) with EGFR mutations following the failure of EGFR-TKI treatment. Recent evidence suggests that similar histological changes can also occur in advanced NSCLC without driver gene mutations after developing resistance to immunotherapy. In this review, it was found that 66.7% of cases with immunotherapy-induced histological transformation were classified as lung squamous cell carcinoma (LSCC), while histological conversion into lung adenocarcinoma (LUAD) without EGFR or ALK gene mutations has rarely been reported. There have been sporadic reports on the occurrence of mutual transformation between LUAD and LSCC. The histological conversion from NSCLC into small cell lung cancer (SCLC) appears to be significantly underestimated, likely due to the infrequency of re-biopsy following the development of immunotherapy resistance. Several studies have reported a close association between the transformation and mutations at TP53 and the RB1 splice site, as well as the loss of an FBXW7 mutation. However, the exact mechanisms underlying this conversion remain unclear. Currently, there is a lack of guidelines for the management of transformed SCLC from NSCLC following immunotherapy, with chemotherapy being the most commonly employed treatment approach.

## Introduction

According to the most recent cancer statistics, lung cancer ranked second in incidence and first in mortality in 2023. Pathologically, lung cancer is divided into non-small cell lung cancer (NSCLC, about 85% of cases) and small cell lung cancer (SCLC, about 15% of cases) (1). Molecular targeted therapy (represented by EGFR-TKI and ALK-TKI) as first-line therapy has significantly improved the survival and prognosis of patients with advanced NSCLC harboring driver gene alterations. Meanwhile, immunotherapy (represented by PD-1/PD-L1 inhibitors) has revolutionized the anticancer treatment for advanced drive gene–negative NSCLC. Nevertheless, acquired drug resistance inevitably occurs in both targeted therapy and immunotherapy, which is a major clinical problem in advanced NSCLC (2, 3).

The mechanisms responsible for EGFR-TKI resistance are primarily the emergence of a second mutation (e.g., T790M mutation) and bypass pathway activation (e.g., MET) (4). The histological transformation of NSCLC into SCLC has been reported as an important mechanism of EGFR-TKI-resistance that occurs in 2%–15% of NSCLC patients after EGFR-TKI failure (5). Re-biopsy is frequently performed in patients with advanced NSCLC after EGFR-TKI resistance. Similarly, approximately 17% of prostate adenocarcinoma patients experience histological conversion into small cell carcinoma upon androgen-deprivation therapy. However, re-biopsy is not routinely carried out in patients with advanced NSCLC upon immunotherapy failure. Recently, there have been reports that immunotherapy resistance is also related to the histological conversion of NSCLC into SCLC. In the present article, we discuss the role of histological transformation in immunotherapy resistance in NSCLC and highlight a potential therapeutic strategy.

## Incidence of histological conversion of NSCLC into SCLC upon immunotherapy

In 2017, Takuma et al. first reported that a patient with advanced NSCLC, who was initially diagnosed with poorly differentiated carcinoma and without EGFR gene mutation, experienced histological transformation into SCLC after immunotherapy resistance to nivolumab (6). Since then, there have been many similar case reports (shown in **Table 1**) (7–17). It is clear from this that increasing attention is paid to the concept of SCLC conversion as a result of immunotherapy in advanced NSCLC.

**Table 1:** Characteristics of the NSCLC cases with histological transformation upon immunotherapy.

Case	Reference	Year/ Gender	Smoking history	ICIs (line)	Histological type		Interval time from ICIs start to SCLC	Treatment after transformation	Survival time after transformation
					Pre-transformation	Post-transformation			
1	Imakita, 2017 (6)	75/M	Yes	Nivo, 3 cycles (2^nd^)	Poorly differentiated NSCLC	SCLC	8 weeks	Amru	2 months
2	Nagasaka, 2017 (7)	71/M	Yes	Nivo, 14 cycles (2^nd^)	LSCC	LUAD	10 months	NA	NA
3	Abdallah, 2018 (8)	65/M	Yes	Nivo, 5 cycles (2^nd^)	LUAD	SCLC	15 weeks	EC	Response to chemotherapy
4	Abdallah, 2018 (8)	68/M	No	Pembro+PTX/CBP, 4 cycles; followed by Pembro, 36 cycles (1^st^)	Poorly differentiated LSCC	SCLC	2 years	EC	>18 months
5	Okeya, 2019 (9)	66/M	Yes	Pembro, 2 cycles (2^nd^)	LUAD	SCLC	5 weeks	EC	5 months
6	Bar, 2019 (10)	70/F	Yes	Nivo, 14 cycles (5^th^)	LSCC with neuroendocrine features	SCLC mixed with LSCC	16 months	Nivo (continued)	9 months
7	Bar, 2019 (10)	75/M	Yes	Nivo, for 6 months (2^nd^)	LSCC with neuroendocrine features	SCLC	7months	EC	13 months
8	Iams, 2019 (11)	67/F	Yes	Nivo, 36 cycles (2^nd^)	LUAD	SCLC	2 weeks	EC	11 months
9	Iams, 2019 (11)	75/F	Yes	Nivo, 33 cycles (2^nd^)	LUAD	SCLC	>2 years	EC	16 months
10	Sehgal, 2020 (12)	60^+^/F	Yes	Nivo, 47 cycles (2^nd^)	Poorly differentiated LSCC	SCLC	21 months	EC	14 months
11	Si X, 2020 (13)	69/M	Yes	Pembro, 22 cycles (1^st^)	LSCC	SCLC	16 months	EC	>6 months
12	Shen Q, 2021 (14)	69/M	Yes	Sintilimab, 4 cycles (1^st^)	LSCC	SCLC	4 months	None	NA
13	Shen Q, 2021 (14)	71/M	Yes	Nivo, 4 cycles (1^st^)	LSCC	SCLC	5 months	EC	4 months
14	Imakita, 2021 (15)	64/M	Yes	Nivo, 23 cycles (1^st^)	LSCC with neuroendocrine features	SCLC	33 months	IC	NA
15	Imakita 2021 (15)	74/F	No	Nivo, 15 cycles (3^rd^)/ Atezo, 8 cycles (4^th^)	LCNEC	SCLC	21 months	Amru	NA
16	Takuma, 2021 (15)	70/M	Yes	Nivo, 15 cycles	LSCC	SCLC	16 months	Etoposide	NA
17	Liu H, 2022 (16)	75/M	Yes	Pembro+PTX/CBP	LSCC	SCLC	17 months	Pembro (Continued)	>12 months
18	Mariniello, 2022 (17)	68/M	Yes	Pembro, 3 cycles (2^nd^)	LUAD	LSCC	2 months	Weekly PTX	About 4 months

Amru, amrubicin; NA, not available; Nivo, nivolumab; LSCC, lung squamous cell cancer; LUAD, lung adenocarcinoma; -, not described; EC, etoposide/carboplatin; PTX, paclitaxel; Pembro, pembrolizumab; LCNEC, large cell neuroendocrine carcinoma; IC, irinotecan/carboplatin; Atezo, atezolizumab.

For advanced lung squamous cell cancer (LSCC), chemotherapy has been the sole treatment due to the lack of available molecular targets. Recently, PD-1/PD-L1 inhibitors have brought new hope to this type of patient. The NSCLC guidelines of the Chinese Society of Clinical Oncology (CSCO) (version 2023) recommends use of a PD-1/PD-L1 inhibitor alone (e.g., atezolizumab or pembrolizumab) and PD-1 inhibitor–containing combination therapy (e.g., nivolumab and sintilimab) as first- and second-line therapies for advanced LSCC, representing the primary foundation of immunotherapy-based therapy.

In the present review, 66.7% of patients (12/18) developed histological conversion from LSCC into SCLC after immunotherapy resistance (in **Table 1**). Among them, there were two cases that were initially diagnosed as LSCC with neuroendocrine features; one then transformed into SCLC and the other transformed into SCLC mixed with LSCC. Except for two patients without a smoking history, the other 10 patients had a history of tobacco smoking, and except for one patient receiving sintilimab therapy, the other 11 patients were receiving nivolumab or pembrolizumab therapy. The median interval time of transformation was 16.4 months, ranging from 4 to 33 months.

Four patients with lung adenocarcinoma (LUAD) who carried no EGFR/ALK mutations histologically evolved into SCLC. These patients had a history of active smoking and received nivolumab or pembrolizumab treatment before transformation into SCLC. The conversion time ranged from 2 weeks to 2 years (**Table 1**).

Of particular concern is the mutual transformation between LUAD and LSCC. Nagasaka et al. reported a case of a man with stage IVA LSCC who had received paclitaxel/carboplatin for four cycles and nivolumab treatment for 10 months. After nivolumab failure, the patient’s re-biopsy revealed a transformation into LUAD. Repeated examination of the previous specimen found no evidence of LUAD (7). In contrast, Mariniello et al. described a patient with recurrent LUAD (harboring BRAF mutation on exon 11 of p.G469A) who had undergone concomitant chemo-radiotherapy and pembrolizumab for three cycles. The disease progressed, and the second biopsy confirmed the histology of LSCC with a retained previous BRAF mutation on exon 11 (p.G469A), strongly indicating histological transformation (17).

Due to the limitation of small scientific reports, the prevalence of transformed SCLC remains unclear in patients with advanced NSCLC who progress after immunotherapy. There are at least two points to be considered. First, there are currently no guidelines that recommend routine re-biopsy when immunotherapy resistance develops in patients with advanced NSCLC (18). In contrast, for patients who receive EGFR-TKI treatment, repeated biopsy is the standard procedure recommended by the NSCLC guideline of the National Comprehensive Cancer Network (NCCN) and CSCO (19, 20). Second, no molecular targets have been clearly defined for the treatment of immunotherapy-resistant advanced NSCLC (18). In contrast, approximately 50% of NSCLC patients who progress after EGFR-TKI failure harbor secondary and concomitant gene mutations, indicating that they have the opportunity to undergo other targeted therapies (e.g., savolitinib for MET mutation) (4). Thus, the real-world frequency of NSCLC-into-SCLC transformation with immunotherapy resistance is likely underestimated (12). The exact frequency of SCLC transformation remains to be validated in future clinical practice.

## Potential mechanisms for histological transformation from NSCLC into SCLC induced by immunotherapy

Histological transformation of lung cancer was first reported in a female NSCLC patient with EGFR exon 19 deletion, who converted into SCLC after gefitinib resistance in 2006 (21). For EGFR-mutant NSCLC transformation into SCLC, there are many responsible additional gene alterations, such as RB1 loss, TP53 mutations, PIK3CA, BRAF, WNK1, and ETV1 mutations, SPP1 upregulation, and REST inactivation (22–29). Notably, the status of RB1 loss and TP53 mutations in EGFR TKI-treated NSCLC have been considered as an important predictor of SCLC transformation (28, 29). However, only recently has the histological transformation of NSCLC into SCLC upon immunotherapy been gradually recognized. Regarding the pathogenesis of the conversion, albeit still uncertain, two kinds of possible transformation mechanisms are proposed.

One is the selection doctrine of combined ingredients, i.e., that the initial tumor comprises NSCLC and SCLC components. The NSCLC cells are decreased or even disappear after immunotherapy, while the immunotherapy-resistant SCLC cells survive as predominant clones. Clinical analysis revealed that the mixed-histology subtype accounted for approximately 5% of all lung cancer (30). Tang et al., performed whole-exome sequencing and microarray profiling in 9 lung cancer patients with mixed histology, and found that the histologic phenotype of lung cancers was possibly determined by the transcriptomic features rather than the genomic characteristics (31). In 2015, the combined SCLC (c-SCLC) as a subtype of SCLC was characterized by an admixture of elements of SCLC with NSCLC, with the incidence of 1%~3% of all SCLC cases (32). Men et al. reported that among 114 cases with c-SCLC, the most common combined component was LSCC (52.6%), followed by LUAD (32.5%) and large cell cancer (11.4%) (33), all of whom were newly-diagnosed and untreated (34).

As indicated in **Table 1**, there were only a few cases where the pre-transformation tumor exhibited neuroendocrine features in histology (e.g., Cases 6–7, 14–15). Among them, only Case 6 had the mixed histology of SCLC and LSCC after transformation, but no such combination existed before transformation (**Table 1**). Of note, no component of SCLC was found in the pre-transformation biopsy from these patients (**Table 1**). A possible reason may be related with the insufficiency of needle biopsy or bronchoscopic lung biopsy, which probably results in the missing SCLC component. However, whether combined-ingredients hypothesis holds true for the histological transformation upon immunotherapy still remains to be confirmed in a large-sample and real-world clinical study.

The other is the transformation doctrine of common precursor, i.e., that NSCLC and SCLC share a common cancer stem cell. NSCLC cells may turn into SCLC under the pressure of immunotherapy. Regarding EGFR-mutant LUAD conversion into SCLC, most oncologists consider EGFR-mutant LUAD and SCLC to originate from the same alveolar type II cells (5). Sehgal et al. found that a patient with poorly differentiated LSCC (Case 10 in **Table 1**) preserved the original genetic alterations after immunotherapy-triggered conversion into SCLC, i.e., TP53 mutation (p. R283fs*62), CDKN2A mutation (R58), SOX2 amplification, and PIK3CA amplification (12). These results support the doctrine of a common precursor rather than the doctrine of two ingredients. More importantly, the second gene mutations, e.g., TP53 mutation, RB1 splice site mutation, and FBXW7 mutation (Arg441Phe) loss, also occur in the histological conversion from immunotherapy-resistant NSCLC into SCLC, as described by Iams et al. (11), Bar et al. (10), and Si et al. (13), respectively. These foundations highly suggest that immunotherapy may remodel the immune milieu, which triggers additional genetic alterations and ultimately contributes to histological transformation. As a result, the second theory of a common precursor seems more convincing. In addition, the specific mechanisms governing mutual conversion between LUAD and LSCC remain unclear due to sporadic reports. Mariniello et al. reported that an NSCLC patient (Case 18 in **Table 1**) had the same BRAF mutation on exon 11 (p. G469A) before and after transformation from LUAD into LSCC after immunotherapy resistance (17), indicating the possibility of a common cell or origin.

**Figure 1:**
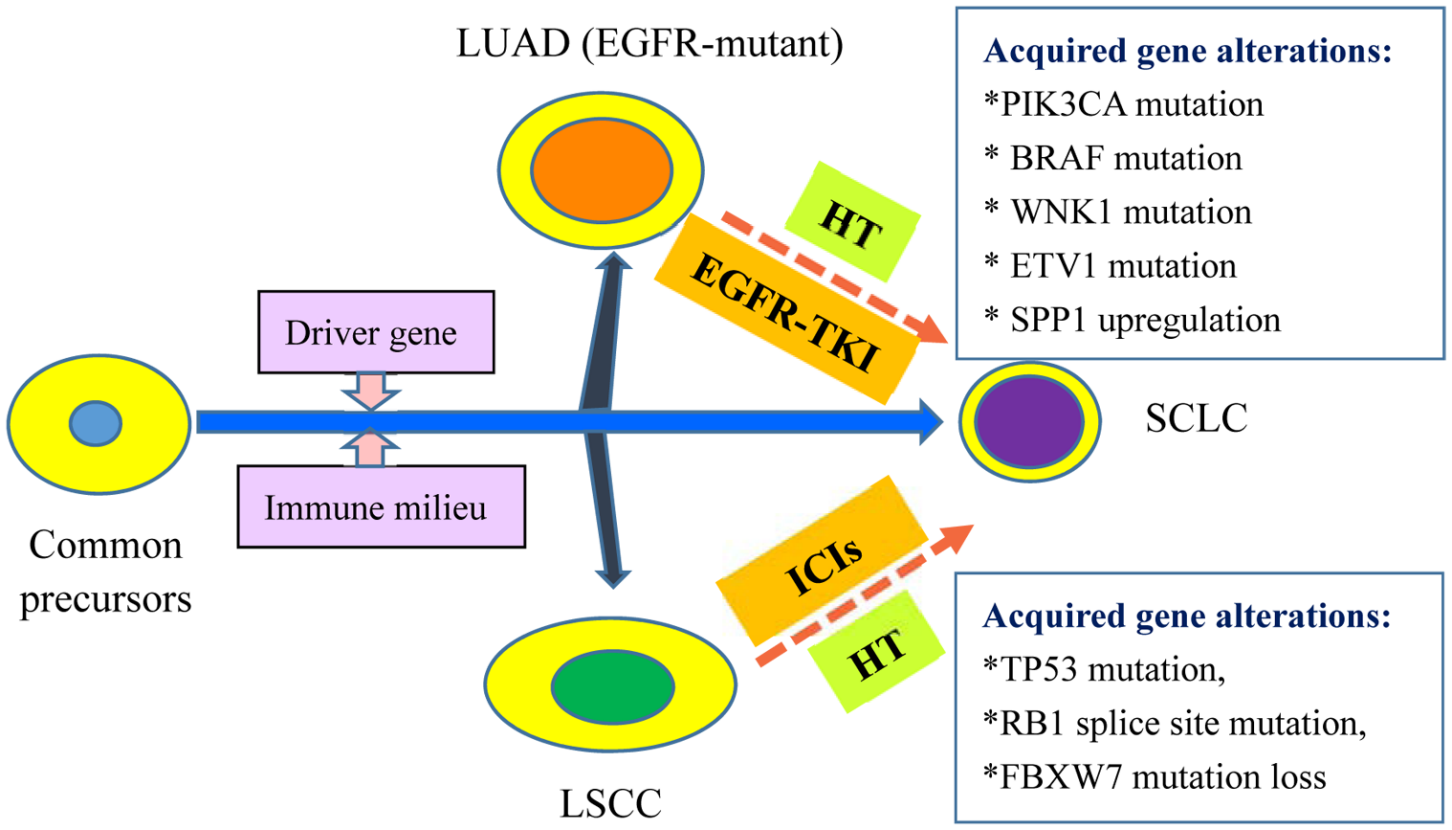
Current Hypothesis of Common Precursors: Histology Transformation to SCLC from NSCLC. The common precursors evolve to LUAD, LSCC and SCLC in different scenarios including driver gene and immune milieu. For EGFR-mutant LUAD treated with EGFR-TKI, acquired gene alterations might be required for the HT to SCLC including *PIK3CA* mutation, *BRAF* mutation, *WNK1* mutation, *ETV1* mutation, *SPP1* upregulation, and *REST* inactivation. For LSCC without available molecular targets, ICIs treatment also result in HT to SCLC. Some acquired gene alterations have been observed in the evolution, including *TP53* mutation, *RB1* splice site mutation and *FBXW7* mutation loss. LUAD, lung adenocarcinoma; EGFR, epidermal growth factor receptor; EGFR-TKI: EGFR- tyrosine kinase inhibitors; HT: histological transformation; LSCC: lung squamous cell cancer; ICIs: immune checkpoint inhibitors; SCLC: small cell lung cancer.

Taken together, most of the literature supports the second doctrine (i.e., common precursor) (**Figure 1**), as the unique phenomenon that transformed SCLC still retains the same gene alterations as the original histology of either LSCC or EGFR-mutant LUAD. However, the potential mechanisms governing the histological conversion, and in particular that for immunotherapy-induced transformation from NSCLC into SCLC, remain to be thoroughly investigated in the future.

## Therapeutic strategies for SCLC transformed from NSCLC after immunotherapy

For extensive-stage *de novo* SCLC, combination chemotherapy with etoposide/carboplatin (EC) has been the standard regimen since last two decades. Since 2019, chemotherapy plus immunotherapy (e.g., a PD-L1 inhibitor, such as atezolizumab, durvalumab, or adebrelimab, and a PD-1 inhibitor, such as serplulimab) has been recommended as the first-line treatment, with median overall survival (mOS) reaching 12.3–15.4 months (34–37). However, there is currently a lack of guidelines for managing SCLC transformed from NSCLC after immunotherapy failure.

According to the literature (**Table 1**), the majority of transformed SCLC cases were treated with combination chemotherapy using EC or irinotecan/carboplatin (IC). The overall survival (OS) after transformation was 11.8 ± 4.51 months, ranging from 6 to 18^+^ months, and the longest OS was more than 18 months. Only a few cases were managed using amurubicin (Cases 1 and 15) or paclitaxel alone (Case 18), but the OS was only approximately 2 to 4 months. The reports indicate that combination chemotherapy achieves better efficacy than single-agent chemotherapy.

The second therapeutic strategy is the continuation of immunotherapy alone. As described in **Table 1**, Cases 6 and 17 received continued treatment of nivolumab or pembrolizumab, and achieved an OS of 9 months and of over 12 months, respectively. In spite of the small sample size, the two reports suggested the potential feasibility of prolonged immunotherapy. However, this needs to be further verified by expanding the sample size.

At present, the therapeutic strategy of antiangiogenesis targeting VEGF has become an indispensable strategy for cancer treatment. In 2019, CSCO recommended anlotinib as the only antiangiogenic agent for refractory extensive-stage *de novo* SCLC in China based on the results of the ALTER 1202 study, which compared the efficacy of anlotinib versus placebo, namely, median progression-free survival (mPFS) (4.1 vs. 0.7 months, P < 0.0001) and mOS (7.3 vs. 4.9 months, P = 0.0029) (38). Furthermore, the ACTION-2 study prospectively reported that the first-line treatment with EP plus anlotinib for extensive-stage *de novo* SCLC achieved an overall response rate (ORR) of 87.2%, a disease control rate (DCR) of 97.7%, an mPFS of 9.0 months, and an mOS of 19.0 months (39). In our retrospective study, transformed SCLC after EGFR-TKI failure was treated with EP plus anlotinib, reaching 9.0 months of mPFS and 14.0 months of mOS (40). At present, there are no reports about anlotinib treatment for the transformed SCLC from NSCLC on immunotherapy. According to the clinical outcome of *de novo* SCLC and EGFR-TKI–induced transformed SCLC, we boldly speculate that the combination regimen of anlotinib with EC chemotherapy may be considered a potential therapeutic strategy. However, this treatment still needs to be tested in real-world clinical practice.

## Conclusions

With the widespread application of immunotherapy, there has been an increasing histological transformation of NSCLC without targetable driver genes. Because re-biopsy is not routinely taken after immunotherapy resistance, the true incidence of histological conversion is almost certainly underestimated in patients with advanced NSCLC, and in particular those with LSCC. There have been a few reports describing the close relation with additional gene alterations, including TP53 mutation, RB1 splice site mutation, and loss of FBXW7 mutation (Arg441Phe). However, this might be just the tip of the iceberg, and the exact mechanism of histological transformation resulting from immunotherapy remains to be clarified. Regarding therapeutic strategy, no guidelines are available for transformed SCLC from immunotherapy-resistant NSCLC. EC chemotherapy is the most common treatment, and the combination of EC with anlotinib may be a potential treatment strategy instead of chemotherapy alone. Of course, the small and insufficient literatures are the primary limitation in our review. We believe that the problem will be gradually resolved with the continuing attention on immunology-induced histological transformation from clinical oncologists.

## Author contributions

JZ: Investigation, Methodology, Writing – original draft. XD: Data curation, Investigation, Writing – original draft. JD: Conceptualization, Data curation, Supervision, Writing – review & editing. XW: Funding acquisition, Supervision, Validation, Writing – review & editing.
